# Personals

**Published:** 1912-12

**Authors:** 


### PERSONALS.

---

Our readers are requested to send notes concerning themselves and others.

---

Dr. Robert R. B. FitzGerald, of Lockport, a graduate of Toronto, 1905, was naturalized, Nov. 9.

---

Dr. Harry Huver of Buffalo and Williamsville, has contracted to administer natural gas to Kenmore.

---

Among the 20 Buffalo delegates to the N. Y. State Conference of Charities and Correction, at Syracuse, Nov. 19-21, were Drs. John H. Pryor and Arthur W. Hurd.

---

Dr. L. M. Andrews of Warsaw has been appointed jail physician for the ensuing year.

---

Dr. J. A. Rafter of North Tonawanda was elected mayor of that city by a large majority.

---

Dr. John H. Grant has moved from Albany to 202 W. 79th St., N. Y.

---

Dr. George S. Staniland has moved to 617 W. Ferry St., Buffalo.

---

Dr. Russell H. Wilcox of N. Tonawanda spent two weeks hunting in Chemung Co., during November.

---

Dr. Walter Calihan has returned to Rochester and opened his office at 170 Clinton Ave., South.

Dr. V. D. Bozovsky of Dunkirk delivered a lecture in the High school Auditorium, Nov. 14, on the Causes of the Present Turkish War. Dr. Bozovsky is a Bulgarian by birth, though a resident of this country for 15 years. One of his brothers is a Major in the Bulgarian Army.

---

The Editor acknowledges the courtesy of the Hon. Simon Seibert in taking him from Gowanda to Perrysburg in time to be present at the dedication of the J. N. Adam Memorial Hospital.

---

Dr. Fenton B. Turck has moved his office from Chicago to 14 East 53rd Street, New York City, Dr. Turck is locating in New York as a director of a research laboratory founded to further the study of the cause and treatment of diseases of the digestive tract.

---

Dr. Warren W. Britt of North Tonawanda, has returned from Chicago, where he attended the convention of the American Medical Association.

---

Dr. Wm. R. Blighton of North Tonawanda, addressed the Legislative Committee of Montpelier, Vt., on "The Compulsory Vaccination Bill." Dr. Blighton spoke in opposition to the bill.

---

Dr. H. W. Bowers and son, of North Tonawanda, have returned from Toronto.

---

Dr. F. X. Fiegel of Tonawanda, has moved to Rosewell, New Mexico. On account of Dr. Fiegel's poor health, a change of climate was necessary.

---

Dr. A. J. Martin of North Tonawanda has returned from a nine's weeks trip to the Pacific coast.

---

Dr. W. E. Dignen of Buffalo has returned from Addison.

---

Dr. William M. Brown of Rochester is making a good recovery from his recent illness.

---

Dr. Anthony Bassler of New York City has been appointed Clinical Professor of Medicine in the N. Y. Polyclinic School and Hospital.

---

Among the Buffalo physicians who attended the Clinical Congress of Surgeons, were Drs. Edith R. Hatch, George F.

Cott, Wm. H. Mansperger, John Fairbairn, Charles Bethune, Marcel Hartwig, Irving W. Potter, Wm. L. Phillips, and Ray Johnson.

---

The following physicians from Elmira attended the Clinical Congress: Drs. Colegrove, Fudge, Booth, Soble and Haase.

---

At the Clinical Congress of Surgeons held in New York City, the following from Rochester were present: Drs. Bascom, Berkman, Bowen, Bissell, Crews, Dickinson, Fowler, Howk, O. E. Jones, W. B. Jones, Remington, Roe, Sanders, Stoddard, Ward and Wickens; also Dr. Fleming of Charlotte, Dr. Hazen of Brockport, Dr. Tinker of Ithaca, and Dr. Roby and Dr. Jewett, who attended the Clinic for Children's Diseases.

---

Dr. Willy Meyer of New York, after demonstrating his apparatus for negative pressure, met Dr. George E. Fell of Buffalo, with whose pioneer work in artificial respiration, he was already familiar. He credited Dr. Fell with laying the foundation for negative pressure apparatus.

---

Dr. Robert P. Bush, of Chemung Co., was relected to the Assembly. Dr. Bush has done good work in leading the fight against legislation which would abolish our present vivisection laws.

---

Drs. Fisher, Baker, Mark, Parke, Voorhees and Haase, of Elmira, attended the Sixth District Branch at Binghamton, on Sept. 15th.

---

Dr. Ira W. Livermore of Gowanda, recently delivered a lecture on the History of the Niagara Frontier, illustrated with stereoptican views.

